# The effect of early remdesivir administration in COVID-19 disease progression in hospitalised patients

**DOI:** 10.1007/s00508-024-02377-7

**Published:** 2024-06-17

**Authors:** Moritz Platzer, David Totschnig, Mario Karolyi, Tamara Clodi-Seitz, Christoph Wenisch, Alexander Zoufaly

**Affiliations:** 1 4. Med. Department for Infectious Diseases and Tropical Medicine, Klinik Favoriten, Wiener Gesundheitsverbund, Vienna, Austria; 2 Faculty of Medicine, Sigmund Freud University, Vienna, Austria

**Keywords:** COVID-19 (omicron & delta), Remdesivir, Timing of therapy, Hospitalized adults, Disease progression

## Abstract

**Background:**

Antiviral drugs have become crucial in managing COVID-19, reducing complications and mortality. Remdesivir has emerged as an effective therapeutic drug for hospitalized patients at risk of disease progression, especially when alternative treatments are infeasible. While the recommended treatment duration of remdesivir extends up to 7 days post-symptom onset, this study examines how early remdesivir administration impacts clinical outcomes.

**Methods:**

We conducted a retrospective analysis using clinical data from consecutively PCR confirmed SARS-CoV‑2 adult patients (≥ 18 years) who received remdesivir during their hospitalization at the department of infectious diseases, Klinik Favoriten in Vienna. The data covered the period from July 1, 2021, to April 31, 2022. Patients were divided into two groups based on the timing of remdesivir administration: an early group (0–3 days since symptom onset) and a late group (≥ 4 days since symptom onset). The primary outcome was in-hospital disease progression, assessed using the WHO COVID-19 Clinical Progression Scale (≥ 1 point increase). Multivariable logistic regression, adjusted for age, sex, SARS-CoV‑2 variant, and COVID-19 vaccination status, was used to assess clinical outcomes.

**Results:**

In total 219 patients were included of whom 148 (67.6%) were in the early group and 71 (32.4%) were in the late group. The average age was 66.5 (SD: 18.0) years, 68.9% of the patients were vaccinated, and 72.6% had the Omicron virus variant. Late remdesivir administration was associated with a significantly higher probability of needing high-flow oxygen therapy (OR 2.52, 95% CI 1.40–4.52, *p* = 0.002) and ICU admission (OR 4.34, 95% CI 1.38–13.67, *p* = 0.012) after adjusting for confounders. In the late group there was a trend towards a higher risk of clinical worsening (OR 2.13, 95% CI 0.98–4.64, *p* = 0.056) and need for any oxygen therapy (OR 1.85, 95% CI 0.94–3.64, *p* = 0.074).

**Conclusion:**

Compared to patients who received remdesivir within the first 3 days after symptom onset, administering remdesivir after day 3 in hospitalized COVID-19 patients is associated with higher risk for complications, such as the need for high-flow oxygen therapy and ICU admission.

**Supplementary Information:**

The online version of this article (10.1007/s00508-024-02377-7) contains supplementary material, which is available to authorized users.

## Introduction

Coronavirus disease 2019 (COVID-19) caused by severe acute respiratory syndrome coronavirus 2 (SARS-CoV-2) emerged as a global pandemic at the beginning of 2020 [1], with older individuals and those with pre-existing conditions such as cardiovascular diseases, immunosuppression, obesity being at higher risk for severe illness and hospitalization [2].

Remdesivir, a ribonucleotide analogue inhibitor of viral RNA polymerase, exhibits broad-spectrum antiviral properties [3]. It has demonstrated early in vitro activity against the Alpha lineage of SARS-CoV‑2 and continues to show in vitro activity against the Omicron variants and its subvariants [4–6].

Currently intravenous remdesivir is approved by the FDA and EMA for the treatment of COVID-19 in both hospitalized patients and non-hospitalized patients at risk for clinical progression to severe disease. Treatment should begin within 7 days of symptom onset and continue for three to five days and can continue up to a maximum of ten days, depending on disease severity and patient’s risk profile [7, 8]. Remdesivir has a low side effect rate in COVID-19 patients, with nausea and liver enzyme elevation being the most common ones [9]. It has recently been approved for treatment in patients with severe kidney failure by the FDA [10].

The current evidence for remdesivir’s efficacy regarding mortality in hospitalized patients with COVID-19 is inconclusive. While some studies have shown favorable outcomes, indicating a decrease in mortality in patients with no or low oxygen need [11–13], other studies showed that remdesivir had little to no effect on mortality [14–16]. Additionally, there is uncertainty regarding the impact of remdesivir on the duration to clinical improvement in terms of oxygen dependency for both invasively and non-invasively ventilated patients [11, 14, 17]. As a result guidelines, such as those from the Infectious Diseases Society of America (IDSA) and the National Institutes of Health (NIH), recommend against the use of remdesivir for mechanically ventilated COVID-19 patients [18, 19]. Other benefits of remdesivir like reducing the risk of disease progression [11] or reducing the time to recovery [17] were shown in earlier disease stages before the need for High Flow Oxygen Therapy. Regarding this, an initiation of remdesivir in an earlier disease stage seems have a more favourable outcome.

Early initiation of antiviral treatment has also shown to improve outcomes and reduce morbidity in other acute viral infections [20, 21]. This therapeutic approach, seems to have beneficial effects for the antiviral treatment of COVID-19 with remdesivir in outpatients as well [22]. In this study we present the findings of a retrospective data analysis conducted during the delta and omicron periods, focusing on examining the influence of the timing of remdesivir administration on the clinical outcomes of hospitalized patients.

## Materials and methods

In this retrospective data analysis, we conducted a thorough screening of all patient records for those who were hospitalized in the department of infectious disesases and tropical medicine, Klinik Favoriten Vienna, between 1st of July 2021 until the 31st of April 2022. In total 1314 patient’s records were screened. The study included adult patients (aged ≥ 18 years) with consecutive positive SARS-CoV‑2 PCR results who received at least one infusion of remdesivir during their hospitalization and had sufficient data available to determine the timing of remdesivir therapy since COVID-19 symptom onset. Patients were ineligible when symptom onset was unknown or data was insufficient to determine symptom onset. The duration of remdesivir therapy, as well as the use of additional antiviral agents such as monoclonal antibodies and supportive drugs like dexamethasone, were determined by the treating physicians in accordance with current guidelines, clinical severity, and the rate of patient improvement. The data analysis was approved by the local ethic committee and was conducted in accordance with the Declaration of Helsinki. Data was obtained anonymously and according to the EU privacy policy.

The collected demographic data consisted of information on sex, age, BMI, COVID-19 vaccination history, and medical history. Additionally, data on the SARS-CoV‑2 variant of concern confirmed by PCR, vital parameters at admission (oxygen saturation, respiratory rate, heart frequency and blood pressure), and daily oxygen saturation during hospitalization were obtained. The study also extracted information on COVID-19 specific therapies and other treatments, including the use of antibiotics.

Symptom onset was defined as the appearance of the first among a list of COVID-19 associated symptoms, including cough, fever, fatigue, myalgia and arthralgia, dyspnea, loss of smell, and gastrointestinal symptoms. The initiation of remdesivir therapy after symptom onset was defined using 24-hour intervals, where day 0 represented the first 24 h since symptom onset (day 0: 0–24 h, day 1: 24–48 h since symptom onset etc.).

**Fig. 1:**
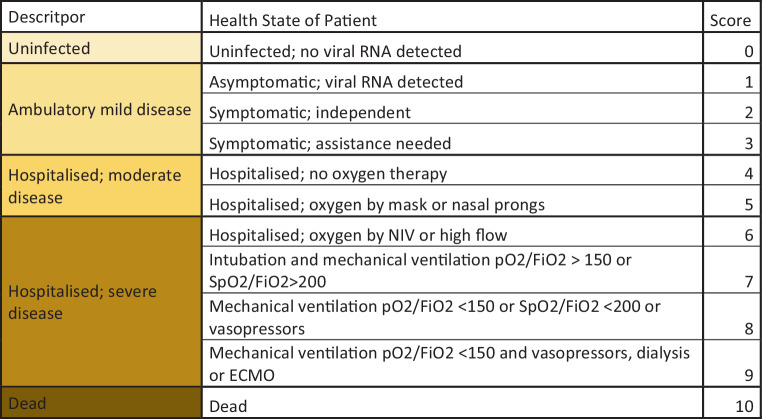
COVID-19 clinical progression scale (Adapted from WHO COVID-19 clinical progression scale [23])

In-hospital disease progression was measured according to the WHO COVID-19 clinical progression scale (Fig. 1; [23]). All patients were assigned a baseline score according to the clinical progression scale at hospital admission. In hospital disease progression was determined by an increase of at least one point compared to baseline. Patients who required Intensive Care Unit attention with mechanical oxygen therapy or medical support with vasopressors and had a score between 7 to 9 on the WHO COVID-19 Clinical Progression Scale were summarized to one progression point. Mortality was assessed as death occurring during hospitalization.

The primary outcome was in hospital diseases progression according the WHO COVID-19 Clinical Progression Scale. Secondary endpoints included any need for oxygen therapy, need for high flow oxygen therapy, ICU admission and in hospital mortality.

Statistical analysis was performed in IBM SPSS Statistic 28.0. For the statistical analysis patients were divided into two groups of early (0–3 days since symptom onset) and late (≥ 4) remdesivir administration. Baseline characteristics were compared using the t‑test or ranksum test for normally or non-normally distributed data and the chi-squared test for categorical variables. Confidence interval was chosen to be 95% and a one-sided *p* < 0.05 was considered significant. Odds ratios for the primary and secondary end points were calculated and adjusted to age in two categories (18–60 years/60+ years), sex (female/male), virus variant (delta/omicron) and vaccination status (at least one COVID-19 vaccination/no vaccine) with multinominal logistic regression. Duration of hospitalization was calculated with independent samples t‑test.

## Results

### Patients

In total 1314 patient’s records were screened. Among all screened patients, 312 patients received remdesivir of which 219 were eligible for study inclusion. The distribution between the early remdesivir initiation group (day 0–3) and late remdesivir initiation group (day 4+) was 148 (67.6%) and 71 (32.4%), respectively. The overall vaccination rate was 68.9%, and a high proportion of the patients had the omicron virus variant (72.6%). These percentages were also comparably high in the early remdesivir initiation group. Diabetes, hypertension, coronary heart disease, malignancy, chronic kidney disease and obesitiy were the most common comorbidities but the number of underlying diseases did not differ between groups. Further demographic characteristics are shown in Table 1.

**Table 1:** Demographic characteristics

Variable	Total		Early (day 0–3)		Late (day 4+)		*p*-Value
	*N* (%)	M (SD)	*N* (%)	M (SD)	*N* (%)	M (SD)	
*Population*	219 (100)	–	148 (67.6)	–	71 (32.4)	–	–
*Sex*							0.432
Male	115 (52.5)	–	75 (50.7)	–	40 (56.3)	–	–
Female	104 (47.5)	–	73 (49.3)	–	31 (43.7)	–	–
*Age (years)*	–	66.5 (18.0)	–	68.0 (18.1)	–	63.5 (17.7)	0.088
18–60	76 (34.7)	–	46 (31.1)	–	30 (42.3)	–	–
60+	143 (65.3)	–	102 (68.9)	–	41 (57.7)	–	–
*BMI*	–	26.7 (6.4)	–	25.7 (5.3)	–	28.9 (8.0)	**<** **0.001**
*Vaccinated against COVID-19*	151 (68.9)	–	117 (79.1)	–	30 (47.6)	–	**<** **0.001**
*Virus variant*							**<** **0.001**
Delta	58 (72.6)	–	23 (15.5)	–	35 (49.3)	–	–
Omikron	159 (58.2)	–	123 (83.1)	–	36 (50.7)	–	–
*Comorbidities*	–	3 (1.9)	–	3 (1.9)	–	3 (1.9)	0.139
Smoker	95 (43.4)	–	67 (45.3)	–	28 (39.4)	–	0.415
Obesitiy	46 (21.0)	–	24 (16.2)	–	22 (31.0)	–	**0.012**
Hypertonia	120 (54.8)	–	85 (57.4)	–	35 (49.3)	–	0.257
Diabetes	63 (28.8)	–	44 (29.7)	–	19 (26.8)	–	0.650
Chronic Kidney Disease	46 (21.0)	–	29 (19.6)	–	17 (23.9)	–	0.460
Coronary Heart Disease	46 (21.0)	–	36 (24.3)	–	10 (14.1)	–	0.082
Atrial Fibrillation	41 (18.7)	–	33 (22.3)	–	8 (11.3)	–	**0.050**
Heart Failure	28 (12.8)	–	20 (13.5)	–	8 (11.3)	–	0.641
Chron. Lungdisease	31 (14.2)	–	19 (12.8)	–	12 (16.9)	–	0.419
Chron. Liverdisease	16 (7.3)	–	11 (7.4)	–	5 (7.0)	–	0.917
Malignancy	51 (23.3)	–	38 (25.7)	–	13 (18.3)	–	0.227
Immunosuppressive State	40 (18.3)	–	32 (21.6)	–	8 (11.3)	–	0.063

### COVID-19 therapy

Among the 219 patients who received remdesivir, the average day of first administration since symptom onset was day 3. A three-day course of remdesivir was given to 48.4% of patients, while 46.1% received a five-day course. Additional COVID-19 therapies included monoclonal antibodies (sotrovimab and regdanvimab), administered to 69 patients (31.5%), dexamethasone given to 87 patients (39.7%) and other immunomodulatory therapeutics such as baricitinib and tocilizumab, given to 21 patients (9.6%). Moreover, 62 patients (28.4%) received antibiotic therapy during hospitalization. A more detailed view of specific therapeutics can be found in Table 2.

**Table 2:** Therapeutical characteristics during hospitalization

Variable	Total		Early (day 0–3)		Late (day 4+)		*p*-Value
	*N* (%)	M (SD)	*N* (%)	M (SD)	*N* (%)	M (SD)	
*Remdesivir administration since symptom onset (days)*	–	3 (3)	–	1 (1)	–	6 (2)	**0.001**
*Remdesivir administration since hospitalisation (days)*	–	0 (1)	–	0 (1)	–	0 (1)	0.637
*WHO Clinical Progression Scale Score at administration*	–	5 (1)	–	4 (1)	–	5 (0)	**0.004**
*Remdesivir therapy after day 7 since symptom onset*	8 (3.7)	–	0 (0)	–	8 (11.3)	–	**–**
*Duration of Remdesivir Therapy*							
1 day	8 (3.7)	–	6 (4.1)	–	2 (2.8)	–	–
3 days	106 (48.4)	–	85 (57.4)	–	21 (29.6)	–	–
5 days	101 (46.1)	–	54 (36.5)	–	47 (66.2)	–	–
Duration unknown	4 (1.8)	–	3 (2.0)	–	1 (1.4)	–	–
*Inpatient therapy*							
Antibiotic Treatment	62 (28.4)	–	43 (29.1)	–	19 (26.8)	–	0.783
Monoclonal Antibody	69 (31.5)	–	42 (28.4)	–	27 (38.0)	–	0.150
Dexamethason	87 (39.7)	–	43 (29.1)	–	44 (62.0)	–	**<** **0.001**
Immunomodulatory therapy other than Dexamtheson	21 (9.6)	–	9 (6.1)	–	12 (16.9)	–	**0.011**

### Outcome

During their hospitalization, 19.2% of patients experienced clinical worsening of their condition according to the WHO COVID-19 Clinical Progression Scale. The late group showed a trend towards an increased probability of clinical deterioration (OR 2.13 95% CI 0.98 to 4.64, *p* = 0.056) and any need for oxygen therapy. However, these results did not reach statistical significance. Patients in the late group had a significant higher probability of receiving high-flow oxygen therapy (OR 2.52 95% CI 1.40 to 4.52, *p* = 0.002) or being admitted to ICU (OR 4.34 95% CI 1.38 to 13.67, *p* = 0.012), after adjusting for age, sex, vaccination status, and viral variant. On average patients were hospitalized for 13 day and in total 15 patients died, without there being a significant difference in mortality. Outcomes are displayed in Table 3.

**Table 3:** Clinical outcome adjusted to age, sex, virus variant and vaccination status

Variable	Total		Early (day 0–3)		Late (day 4+)		OR (95% CI)	*p*-Value
	*N* (%)	M (SD)	*N* (%)	M (SD)	*N* (%)	M (SD)		
*Clinical Progression*	42 (19.2)	–	21 (14.2)	–	21 (29.6)	–	2.13 (0.98–4.64)	0.056
*Any Need for Oxygen Therapy*	111 (50.7)	–	65 (43.9)	–	46 (64.8)	–	1.85 (0.94–3.64)	0.074
*High Flow Oxygen* *Therapy*	27 (12.3)	–	8 (5.4)	–	19 (26.8)	–	4.68 (1.76–12.44)	**0.02**
*ICU Admission*	20 (9.1)	–	8 (5.4)	–	12 (16.9)	–	4.34 (1.38–13.67)	**0.012**
*Death*	15 (6.8)	–	13 (8.8)	–	2 (2.8)	–	0.32 (0.66–1.53)	0.153
*Duration of* *Hospitalisation (Days)*	–	13 (8)	–	13 (9)	–	12 (7)	–	0.051

## Discussion

In this retrospective single-center data analysis including hospitalized PCR confirmed COVID-19 patients during the delta and omicron wave, who received remdesivir for treatment, early remdesivir administration was associated with a significantly lower probability for need of high flow oxygen therapy and a lower risk for ICU admission, after adjusting for age, sex, vaccination status, and viral variant. Patients who received remdesivir on day 4 or later after first symptoms had a 2–3 time higher chance to need high flow oxygen therapy and a more than 4‑fold higher risk to be admitted to the ICU. Furthermore, the risk for clinical progression seemed to have doubled in patients who received remdesivir after day 4 of symptom onset and there was a trend for an increased need for any oxygen therapy.

The relationship between early administration of remdesivir and a favourable clinical outcome of hospitalized patients has previously been shown in randomized trials. Beigel et al. showed that patients who received remdesivir within the initial 10 days after symptom onset had a higher rate ratio for recovery, than those who received it after the first 10 days, suggesting a more beneficial outcome when remdesivir was administered early [17]. A retrospective multi-center study from Spain suggested that the administration of remdesivir in the first five days was associated with a lower probability of ICU admission [24]. The meta-analysis conducted by the WHO Solidarity Trial Consortium indicated a more favourable outcome when remdesivir was administered during the early stages of disease progression, in patients who did not require supplemental oxygen or only needed low flow oxygen [11], also suggesting a more favourable outcome when started early.

In non-hospitalized PCR confirmed SARS-CoV‑2 patients with risk of disease progression Gottlieb et al. showed that early (median was day 5 since symptom onset) outpatient treatment with remdesivir was linked to a significant reduction in hospitalization or death [22]. Rajme-Lopez et al. were able to demonstrate similar results when remdesivir was given early (median was day 3 since symptom onset) for outpatient treatment [25].

To our knowledge, this is the first study that has undertaken an analysis of the timing of remdesivir administration in hospitalized patients during both the delta and omicron waves. The early initiation of remdesivir treatment is linked to better outcomes, while administering it at a later stage in disease appears to be associated with a higher likelihood of unfavourable outcomes. This could be attributed to delayed hospitalization, where patients who arrive later may already be in an advanced disease stage, resulting in inferior results. Moreover, these patients may not have been provided with the option of earlier outpatient remdesivir treatment due to potential logistical challenges associated with outpatient intravenous administration of the drug.

This becomes particularly crucial when oral nirmatrelvir/ritonavir is contraindicated due to organ failure, potential drug interactions, symptoms persistence for more than five days or need for hospitalization. Patients at risk of disease progression often take medications that interact with nirmatrelvir/ritonavir [18]. Thus making the prescription process challenging. For instance, organ transplant recipients under immunosuppressants like tacrolimus require close monitoring by specialists if considering nirmatrelvir/ritonavir for SARS-CoV‑2 [26]. If nirmatrelvir/ritonavir is not feasible, treatment with intravenous remdesivir is a viable alternative with fewer drug interactions. Therefore, the identification of high-risk individuals and the prompt initiation of remdesivir are of significant importance in hospitalized and outpatient settings.

However, physicians might be more reluctant to prescribe remdesivir as early as possible, because even if intravenous remdesivir can be stored at room temperature and does not require special infusion settings, its administration might be challenging in certain healthcare settings as providing consecutive intravenous doses over 3 to 5 days could pose logistical difficulties. A viable alternative for an early treatment start might be oral remdesivir agents. For example VV116, a deuterated remdesivir hydrobromide with oral bioavailability, has shown non inferiority to nirmatrelvir–ritonavir to the time to sustained clinical recovery, with fewer safety concerns [27].

### Limitations

This study possesses several limitations due to its retrospective nature, including data incompleteness and potential recruitment biases, which may introduce unknown confounders. Additionally, the absence of a control group not receiving remdesivir restricts the conclusions to the comparison between early and late remdesivir administration, making it challenging to evaluate remdesivir’s overall efficacy in COVID-19 treatment. Moreover there was an overrepresentation of the early group with a higher proportion in Omicron cases and vaccinated individuals, both associated with milder disease progression. In addition the late group scored higher on the WHO Clinical progression scale upon admission, suggesting a poorer health status at baseline. These factors may have contributed to a confounding bias in the analysis, despite attempts to adjust the data for vaccination status and virus variant. Furthermore, data was collected during the Delta and early Omicron waves, whereas the current predominant variant is Omicron XBB. In addition, this was a single-center study conducted in Vienna, and results may vary in other settings and countries. Since this study exclusively examined hospitalized patients, there is a potential for an overrepresentation of sicker individuals, which could have influenced the observed outcomes, possibly leading to a less favourable overall outcome.

## Conclusions

Our findings indicate that the early administration (0–3 days since symptom onset) of remdesivir in hospitalized patients may decrease the risk of COVID-19 complications, such as the need for high-flow oxygen therapy and ICU admission. This suggests that patients at a higher risk of developing severe disease, who are unable to take oral antiviral options, should receive remdesivir therapy as early as possible.

### Supplementary Information

**Supplementary data.** Two subanalyses have been conducted based on the collected data. The first compares the clinical outcomes of hospitalized patients in our department who received remdesivir, categorized by virus variants (Delta and Omicron). The second examines the clinical outcomes of hospitalized patients who received remdesivir, categorized by vaccination status. Note that these clinical outcomes were not adjusted for any variables. Please feel free to contact the corresponding author for additional information.

## Data Availability

The data referred to during the study are available from the corresponding author on reasonable request.
