# Supplementary material for: The effect of early remdesivir administration in COVID-19 disease progression in hospitalised patients
Source: Wien Klin Wochenschr. 2024 Jun 17;136(15-16):458–64. doi: 10.1007/s00508-024-02377-7 (PMC11327179; doi:10.1007/s00508-024-02377-7)
Supplement: Supplementary file 1 — Supplementary data. Two subanalyses have been conducted based on the collected data. The first compares the clinical outcomes of hospitalized patients in our department who received remdesivir, categorized by virus variants (Delta and Omicron). The second examines the clinical outcomes of hospitalized patients who received remdesivir, categorized by vaccination status. Note that these clinical outcomes were not adjusted for any variables. Please feel free to contact the corresponding author for additional information. [file 508_2024_2377_MOESM1_ESM.docx]

| VARIABLE | TOTAL | | OMIKRON | | DELTA | | p-VALUE |
| --- | --- | --- | --- | --- | --- | --- | --- |
|  | **N (%)** | **M (SD)** | **N (%)** | **M (SD)** | **N (%)** | **M (SD)** |  |
| Population | 217 (100) |  | 159 (67,6) |  | 58 (32,4) |  |  |
| Sex |  |  |  |  |  |  | 0,244 |
| Male | 113 (52,1) |  | 79 (49,7) |  | 34 (58,6) |  |  |
| Female | 104 (47,9) |  | 80 (50,3) |  | 24 (41,4) |  |  |
| Age (years) |  | 66,5 (18,1) |  | 68,2 (17,7) |  | 61,5 (18,6) | **0,024** |
| 18-60 | 76 (35,0) |  | 46 (31,1) |  | 31 (53,4) |  |  |
| 60+ | 141 (65,) |  | 102 (68,9) |  | 27 (46,6) |  |  |
| Vaccinated against COVID-19 | 150 (68,9) |  | 125 (78,6) |  | 25 (43,1) |  | **<0,001** |
| Comorbidities |  | 3 (2) |  | 3 (2) |  | 2 (2) | **0,003** |

**OMIKRON & DELTA SUBANALYSIS**

| VARIABLE | TOTAL | | OMIKRON | | DELTA | | p-VALUE |
| --- | --- | --- | --- | --- | --- | --- | --- |
|  | **N (%)** | **M (SD)** | **N (%)** | **M (SD)** | **N (%)** | **M (SD)** |  |
| Remdesivir administration since symptom onset (days) |  | 3 (3) |  | 3 (3) |  | 4 (2) | **<0,001** |
| Remdesivir early | 146 (67,3) |  | 123 (77,4) |  | 23 (39,7) |  |  |
| Remdesivir late | 71 (32,7) |  | 36 (22,6) |  | 35 (60,3) |  |  |
| WHO Clinical Progression Scale Score at administration |  | 5 (1) |  | 5 (1) |  | 5 (0) | **0,004** |
| Duration of Remdesivir  Therapy |  |  |  |  |  |  |  |
| 3 days | 104 (47,9) |  | 96 (34,6) |  | 23 (39,7) |  |  |
| 5 days | 101 (46,5) |  | 55 (60,4) |  | 35 (60,3) |  |  |
| Inpatient therapy |  |  |  |  |  |  |  |
| Monoclonal Antibody | 68 (31,3) |  | 50 (31,4) |  | 18 (31,0) |  |  |
| Dexamethason | 87 (40,1) |  | 53 (33,3) |  | 34 (58,6) |  |  |
| Immunomodulatory therapy other than Dexamtheson | 21 (9,7) |  | 9 (5,7) |  | 12 (20,7) |  |  |

| VARIABLE | TOTAL | | OMIKRON | | DELTA | | OR (95% CI) | p-VALUE |
| --- | --- | --- | --- | --- | --- | --- | --- | --- |
|  | **N (%)** | **M (SD)** | **N (%)** | **M (SD)** | **N (%)** | **M (SD)** |  |  |
| Clinical Progression | 42 (19,4) |  | 25 (15,7) |  | 17 (29,3) |  | 0,45 (0,22-0,91) | **0,025** |
| Any Need for Oxygen Therapy | 111 (51,2) |  | 72 (45,3) |  | 39 (67,2) |  | 0,40 (0,22 – 0,76) | **0,007** |
| High Flow Oxygen  Therapy | 27 (12,4) |  | 13 (8,2) |  | 14 (24,1) |  | 0,27 (0,12-0,65) | **<0,001** |
| ICU Admission | 20 (9,2) |  | 14 (8,8) |  | 6 (10,3) |  | 0,84 (0,30-2,29) | 0,729 |
| Death | 15 (6,9) |  | 11 (6,9) |  | 4 (6,9) |  | 1,00 (0,36-3,29) | 0,996 |
| Duration of  Hospitalisation (Days) |  | 13 (8) |  | 13 (8) |  | 13 (10) |  |  |

**VACCINATION STATUS SUBANALYSIS**

| VARIABLE | TOTAL | | VACCINATED | | UNVACCINATED | | p-VALUE |
| --- | --- | --- | --- | --- | --- | --- | --- |
|  | **N (%)** | **M (SD)** | **N (%)** | **M (SD)** | **N (%)** | **M (SD)** |  |
| Population | 217 (100) |  | 151 (69,6) |  | 66 (30,4) |  |  |
| Sex |  |  |  |  |  |  | 0,320 |
| Male | 113 (52,1) |  | 82 (45,7) |  | 35 (53,0) |  |  |
| Female | 104 (47,9) |  | 69 (54,3) |  | 21 (47,0) |  |  |
| Age (years) |  | 66,5 (18,1) |  | 67,7 (17,7) |  | 63,7 (18,8) | 0,135 |
| 18-60 | 75 (34,6) |  | 46 (30,5) |  | 31 (53,4) |  |  |
| 60+ | 142 (65,4) |  | 105 (69,5) |  | 27 (46,6) |  |  |
| Virus variant |  |  |  |  |  |  | **<0,001** |
| Omikron | 158 (72,8) |  | 125 (82,8) |  | 33 (50,0) |  |  |
| Delta | 57 (26,2) |  | 25 (16,6) |  | 32 (48,5) |  |  |
| Comorbidities |  | 3 (2) |  | 3 (2) |  | 2 (2) | **0,013** |

| VARIABLE | TOTAL | | VACCINATED | | UNVACCINATED | | p-VALUE |
| --- | --- | --- | --- | --- | --- | --- | --- |
|  | **N (%)** | **M (SD)** | **N (%)** | **M (SD)** | **N (%)** | **M (SD)** |  |
| Remdesivir administration since symptom onset (days) |  | 3 (3) |  | 2 (2) |  | 4 (3) | **<0,001** |
| Remdesivir early | 146 (67,3) |  | 117 (77,5) |  | 29 (43,9) |  |  |
| Remdesivir late | 71 (32,7) |  | 34 (22,5) |  | 37 (56,1) |  |  |
| WHO Clinical Progression Scale Score at administration |  | 5 (1) |  | 5 (1) |  | 5 (0) | **0,004** |
| Duration of Remdesivir  Therapy |  |  |  |  |  |  |  |
| 3 days | 106 (47,9) |  | 82 (54,3) |  | 24 (36,4) |  |  |
| 5 days | 99 (46,5) |  | 59 (39,1) |  | 40 (60,6) |  |  |
| Inpatient therapy |  |  |  |  |  |  |  |
| Monoclonal Antibody | 68 (31,3) |  | 26 (17,2) |  | 42 (63,6) |  |  |
| Dexamethason | 87 (40,1) |  | 49 (32,5) |  | 38 (57,6) |  |  |
| Immunomodulatory therapy other than Dexamtheson | 21 (9,7) |  | 7 (4,6) |  | 14 (21,2) |  |  |

| VARIABLE | TOTAL | | VACCINATED | | UNVACCINATED | | OR (95% CI) | p-VALUE |
| --- | --- | --- | --- | --- | --- | --- | --- | --- |
|  | **N (%)** | **M (SD)** | **N (%)** | **M (SD)** | **N (%)** | **M (SD)** |  |  |
| Clinical Progression | 41 (18,9) |  | 25 (16,6) |  | 16 (24,2) |  | 0,62 (0,31-1,26) | 0,183 |
| Any Need for Oxygen Therapy | 109 (50,2) |  | 68 (45,0) |  | 41 (62,1) |  | 0,50 (0,28 – 0,90) | **0,021** |
| High Flow Oxygen  Therapy | 27 (12,4) |  | 13 (8,6) |  | 14 (21,2) |  | 0,35 (0,16-0,80) | **0,011** |
| ICU Admission | 19 (8,8) |  | 10 (6,6) |  | 9 (13,6) |  | 0,45 (0,17-1,16) | 0,09 |
| Death | 15 (6,9) |  | 12 (7,9) |  | 3 (4,5) |  | 1,81 (0,49-6,65) | 0,363 |
| Duration of  Hospitalisation (Days) |  | 13 (8) |  | 13 (8) |  | 14 (9) |  | 0,367 |
